# Exogenous Trehalose Assists *Zygosaccharomyces rouxii* in Resisting High-Temperature Stress Mainly by Activating Genes Rather than Entering Metabolism

**DOI:** 10.3390/jof10120842

**Published:** 2024-12-05

**Authors:** Xiong Xiao, Quan Liu, Qian Zhang, Zhenzhen Yan, Dongbo Cai, Xin Li

**Affiliations:** 1 Cooperative Innovation Center of Industrial Fermentation (Ministry of Education and Hubei Province), Key Laboratory of Fermentation Engineering (Ministry of Education), Hubei Key Laboratory of Industrial Microbiology, School of Life and Health Sciences, Hubei University of Technology, Wuhan 430068, China; 102200529@hbut.edu.cn (X.X.); 15871452919@163.com (Q.L.); 18095010330@163.com (Q.Z.); 13781684121@163.com (Z.Y.); 2 State Key Laboratory of Biocatalysis and Enzyme Engineering, School of Life Sciences, Hubei University, Wuhan 430068, China; caidongbo@hubu.edu.cn

**Keywords:** *Zygosaccharomyces rouxii*, trehalose, high-temperature adversity, proliferation, transcriptome

## Abstract

*Zygosaccharomyces rouxii* is a typical aroma-producing yeast in food brewing, but it has low heat resistance and poor proliferation ability at high temperature. Trehalose is generally considered to be a protective agent that helps stable yeast cells resist heat shock stress, but its functional mechanism for yeast cells in the adaptation period under heat stress is unclear. In this study, the physiological metabolism changes, specific gene transcription expression characteristics, and transcriptome differences of *Z. rouxii* under different carbon sources under high-temperature stress (40 °C) were compared to explore the mechanism of trehalose inducing *Z. rouxii* to recover and proliferate under high-temperature stress during the adaptation period. The results showed that high concentration of trehalose (20% Tre) could not be used as the main carbon source for the proliferation of *Z. rouxii* under long-term high-temperature stress, but it helped to maintain the stability of the cell population. The intracellular trehalose of *Z. rouxii* was mainly derived from the synthesis and metabolism of intracellular glucose, and the extracellular acetic acid concentration showed an upward trend with the improvement of yeast growth. A high concentration of trehalose (20% Tre) can promote the expression of high glucose receptor gene *GRT2* (12.0-fold) and induce the up-regulation of *HSF1* (27.1-fold), *MSN4* (58.9-fold), *HXK1* (8.3-fold), and other signal transduction protein genes, and the increase of trehalose concentration will maintain the temporal up-regulation of these genes. Transcriptome analysis showed that trehalose concentration and the presence of glucose had a significant effect on the gene expression of *Z. rouxii* under high-temperature stress. In summary, trehalose assists *Z. rouxii* in adapting to high temperature by changing gene expression levels, and assists *Z. rouxii* in absorbing glucose to achieve cell proliferation.

## 1. Introduction

*Zygosaccharomyces rouxii*, as a yeast strain that can tolerate a high-salt environment and has the ability to produce alcohol, occupies an important application position in the traditional industrial food fermentation process [1,2]. However, *Z. rouxii* is less tolerant to high temperature and proliferates slowly at high temperature [3,4]. Studies have shown that some specific strains of *Z. rouxii*, such as strain AS 2.1521, have no signs of growth after 168 h of culture in microplates at 40 °C [5]; the survival rate of strain CGMCC 3791 decreased by 18% after 90 min of high-temperature treatment at 40 °C [6]. The heat tolerance limitation of *Z. rouxii* undoubtedly limits its application potential in specific high-temperature fermentation processes to a certain extent. Therefore, it is of great significance to explore the response mechanism of *Z. rouxii* to high-temperature stress to improve its heat tolerance.

Trehalose (Tre) is a non-reducing disaccharide that is widely considered a natural protective agent [7,8,9]. It can not only be used as a microbial reserve carbon source, but also has the functions of protecting yeast intracellular DNA and stabilizing protein conformation, which plays an important role in resisting heat shock stress [10,11]. A large number of studies on *Saccharomyces cerevisiae* have shown that trehalose protects yeast cells during the stable period [12], which is also manifested in *Kluyveromyces marxianus* [13] and *Z. rouxii* [14]. It is worth noting that studies have shown that trehalose is a key factor in the recovery of *S. cerevisiae* growth in the resting state [15], suggesting that trehalose also plays an important role in the adaptation period of yeast. Therefore, it is necessary to explore the mechanism of trehalose assisting *Z. rouxii* to restore proliferation during the adaptation period under high-temperature stress.

In view of the universality of high-temperature stress in industrial production and its adverse effects on yeast, many scholars have carried out in-depth exploration on the complex mechanism of yeast high-temperature resistance [16]. At present, most of the related research focuses on the tolerance of yeast to short-term heat shock, aiming to reveal the rapid adaptation mechanism of yeast under short-term heat shock [17,18,19]. When *S. cerevisiae* strain BY4741 was cultured at 38 °C, its organelle structure changed significantly within 90 min [20]. When *S. cerevisiae* strain R1158 was exposed to mild heat shock at 37 °C, the change of gene expression peaked in the first 15 min [21]. Although short-term heat shock research can map the adaptation strategy of yeast under long-term heat stress to a certain extent, the impact of time cannot be ignored. *Candida albicans* strain ML250 can activate different kinase signaling pathways to cope with different durations of high-temperature stimulation, which also indicates that microorganisms adopt different coping strategies in the face of high-temperature stress with different durations [22]. Therefore, exploring how *Z. rouxii* adjusts its proliferation strategy under long-term high-temperature stress is not only a necessary supplement to short-term heat shock research, but also the key to understanding the viability of the yeast cells.

Therefore, in this study, *Z. rouxii* was used as the research object. Based on the total synthetic medium under oligotrophic conditions, the growth, metabolic characteristics, and related gene expression differences of *Z. rouxii* under different nutritional conditions under long-term high-temperature stress were investigated. Combined with transcriptomics, the mechanism of trehalose assisting *Z. rouxii* to restore proliferation during the adaptation period under long-term high-temperature stress was revealed, and the understanding of the limited proliferation ability of *Z. rouxii* under high-temperature stress was deepened.

## 2. Materials and Methods

### 2.1. Strains, Medium, and Culture Conditions

*Z. rouxii* was isolated from traditional Chinese brewed food in a laboratory, and the patent number was CTCC M 2013310.

Seed culture medium: YEPD agar medium. The cells were pre-cultured at 30 °C for 1~2 days.

Yeast culture medium: 2% trehalose/20% trehalose/20% trehalose (Tre) + 2% glucose (Glc), 0.4% proline, 0.1% potassium dihydrogen phosphate, 0.05% magnesium sulfate, 1.0 mg/L folic acid, 1.0 mg/L calcium pantothenate. The initial inoculation amount of OD600 after inoculation was maintained at 0.5, at 40 °C and 200 rpm in constant-temperature culture.

### 2.2. Determination of Biomass

Cell optical density or viable cell plate colony count was used as an indicator of biomass. Determination of optical density by turbidity method (OD600 nm): 0.5 mL of yeast suspension was diluted and determined by colorimetric method at a wavelength of 600 nm with deionized water as a control. Plate colony count (CFU): 0.5 mL yeast suspension was diluted and spread on YEPD agar plate and cultured at 30 °C for 48 h.

### 2.3. Detection of Carbon Sources and Metabolites (Glucose, Trehalose, Xylitol, Glycerol)

A 1 mL yeast suspension was centrifuged at 8000 r/min for 5 min at −4 °C, and the supernatant (extracellular carbon metabolites) and yeast (intracellular carbon metabolites) were separated. Of the supernatant filtrated through the cellulose acetate membrane (diameter 13 mm; 0.2 μm), 400 μL was used for the detection of extracellular carbon metabolites by high-performance liquid chromatography (LC-2050, Shimadzu, Kyoto, Japan). After the yeast was quenched with liquid nitrogen, 2 mL of 40% ethanol and glass beads (wet cell/glass beads 1/5, *w*/*w*) were added to a vortex shake for 20 min and centrifuged at 12,000 r/min for 5 min, and 400 μL of supernatant was taken for freeze-drying. The freeze-dried sample was dissolved in 200 μL pyridine (20 mg/mL) for salinization derivatization reaction [23]. At the same time, 100 μL L-ketoglutaric acid (1 mg/mL) was taken and derivatized separately. After derivatization, the sample and L-ketoglutaric acid were mixed at 1:1 (*v*/*v*) and centrifuged at 8000 r/min for 1 min, and 400 μL was used for Agilent high-performance gas chromatography (7890B, Agilent, Santa Clara, CA, USA) to detect intracellular carbon metabolites.

High-performance liquid chromatographic conditions: The column temperature was set at 50 °C, 0.1% (*v*/*v*) sulfuric acid was used as eluent, the flow rate was 1.0 mL/min, and the detector was RID (refractive index detector). The SUGAR SH1011 chromatographic column (8.0 mmID × 300 mmL, Shodex, Kyoto, Japan) was used for detection.

Agilent high-performance gas chromatographic conditions: The detector is a hydrogen flame ion detector; nitrogen was used as carrier gas. A DB-5 capillary column (30 m × 0.32 mm × 0.25 μm, Agilent, Santa Clara, CA, USA) was used; the column temperature was set as follows: after maintaining at 70 °C for 5 min, the column temperature was raised to 280 °C at 5 °C/min, and maintained for 5 min. The injection port was set at 280 °C, and the detector was set at 300 °C.

### 2.4. Detection of Organic Acids (Acetic Acid, Malic Acid)

A 1 mL yeast suspension was centrifuged at 8000 r/min for 5 min at −4 °C, and the supernatant (extracellular organic acid) and yeast (intracellular organic acid) were separated. The supernatant was mixed with an equal volume of pure ethanol and centrifuged at 12,000 r/min for 5 min again. After protein precipitation, the extracellular organic acids were measured by Agilent gas chromatograph (7890B). After the yeast was quenched with liquid nitrogen, 2 mL 40% ethanol and glass beads (wet yeast/glass beads were 1/5, *w*/*w*) were added for vortex oscillation for 20 min and centrifuged at 12,000 r/min for 5 min, and the supernatant was retained. After protein sedimentation, the extracellular organic acids were measured by Agilent gas chromatograph (7890B).

Protein precipitation step: 400 μL of sample was added to 1.6 mL of methanol and centrifuged at 12,000 r/min for 5 min, and the supernatant was collected. Then, 550 μL of supernatant was added with 400 μL of tert-amyl alcohol (0.04%, 65% ethanol as solvent) and 50 μL of formic acid.

Chromatograph conditions: The detector is a hydrogen flame ion detector; nitrogen was used as carrier gas. An HA-INNOWax capillary column (30 m × 0.32 mm × 0.25 μm, Agilent) was used; the column temperature was set as follows: 40 °C for 5 min, then increased 20 °C/min to 120 °C, and then increased 10 °C/min to 220 °C, and maintained for 3 min. The injection port was set at 250 °C, the detector at 280 °C.

### 2.5. Transcriptional Sequencing of Z. rouxii

We collected 200–300 mg of *Z. rouxii* cultured for 0 h, 24 h, and 48 h in 2 mL EP tubes, while *Z. rouxii* cells cultured for 0 h were used as control (named CK). The sampling points of 2% Tre conditions were named Zro_1 and Zro_2, those of 20% Tre conditions were named Zro_3 and Zro_4, and those of 20% Tre + 2% Glc conditions were named Zro_5 and Zro_6. The sequencing samples were taken for three biological replicates. Illumina NovaSeq 6000 sequencing technology was used to complete the processing of sequencing samples in Shanghai Meiji Biomedical Technology Co. Ltd., Shanghai, China [24]. Through screening and cleaning steps, high-quality clean reads were extracted from the original data, and differentially expressed genes were further identified. The enrichment analysis of differentially expressed genes was performed using RSEM (RNA-Seq by Expectation-Maximization, Version 1.3.3) software.

### 2.6. Real-Time Fluorescence Quantitative Detection

The wet yeast cells (200 mg) cultured at 0 h, 12 h, 24 h, and 48 h were collected in 2 mL EP tubes, and the *Z. rouxii* cells at 0 h were used as control. After the total RNA of the yeast was extracted by Tiangen total RNA extraction kit (TIANGEN, Beijing, China), the concentration and purity of the RNA were detected by microspectrophotometer. The cDNA was obtained by using the Vazyme R223-01 reverse transcription kit (Vazyme, Nanjing, China). The PCR conditions were 15 min at 50 °C and 5 s at 85 °C. The Vazyme Q711 kit (Vazyme, Nanjing, China) was used to perform RT-qPCR experiments on the Quant Studio 3 real-time fluorescence quantitative PCR system. The reaction conditions of the polyenzyme chain reaction system reported by Yan et al. [25] were used. ΔCT (Delta cycle threshold) = CT (target gene) value − CT (housekeeping gene, ENO1); −ΔΔCT = CT (control sample) − ΔCT (Delta cycle threshold). Taking the gene transcription expression level of *Z. rouxii* cells at 0 h as a reference, the fold change of the transcription expression level of the target gene was calculated by 2 ^−∆∆CT^ [26]. Primer design: The test-related gene ID was screened from the transcriptome data of *Z. rouxii* M 2013310, and its nucleotide sequence was obtained by the NCBI (National Center for Biotechnology Information, Bethesda, MD, USA). With the help of Primer Premier 6, primer pairs were designed for the nucleotide sequence of the target gene retrieved from NCBI (note: primer design results are detailed in Table S1).

### 2.7. Statistical Methods

All chart data were three biological replicates. Statistical analysis was performed using Excel 2003 (calculating the average value and determining SD). GraphPad Prism9 and GraphPad Origin 2019b software were used to draw graphs. IBM SPSS Statistics 23.0 software (SPSS Inc., Chicago, IL, USA) was used to determine the significant difference of variance analysis, with **** denoting *p* < 0.0001.

## 3. Results and Analysis

### 3.1. Effects of Different Carbon Sources on the Growth of Z. rouxii at High Temperature

**Figure 1:**
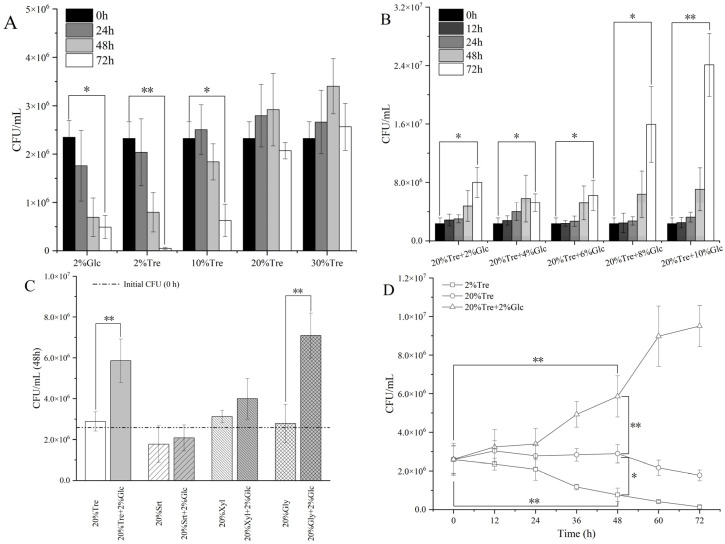
Effects of carbon source concentration and ratio on high-temperature (40 °C) growth of *Z. rouxii:* (**A**) the growth of *Z. rouxii* with 2% Glc and different Tre concentrations; (**B**) the growth of *Z. rouxii* under different concentrations of Glc and 20% Tre; (**C**) the growth of *Z. rouxii* under different osmotic conditions; (**D**) analysis of growth period of *Z. rouxii*. Abbreviations: Tre, trehalose; Srt, sorbitol; Xyl, xylitol; Gly, glycerol. Note: significance test: * *p* < 0.05; ** *p* < 0.01.

Studies have shown that 2% glucose is not enough to maintain the survival of *Z. rouxii* at 40 °C. Trehalose is both a carbon source and a heat protector, which may help *Z. rouxii* to resume proliferation at high temperatures. The growth status of *Z. rouxii* under different carbon sources is shown in Figure 1. As shown in Figure 1A, *Z. rouxii* showed a rapid decline trend when only 2% Tre was used as the carbon source. With the increase of trehalose concentration, the growth decline trend of *Z. rouxii* was significantly slowed down. When the concentration of Tre reached 20%, the survival of *Z. rouxii* could be basically maintained, but it still could not restore its proliferation. This prompted us to continue to explore different carbon sources and their combinations. Subsequently, we added different concentrations of glucose on the basis of 20% Tre. As shown in Figure 1B, *Z. rouxii* was able to restore proliferation when only 2% glucose was added. In order to determine whether the recovery of growth is caused by osmotic stress, we also evaluated the effects of different osmotic agents on the growth of *Z. rouxii.* As shown in Figure 1C, at 48 h, except trehalose, only the combination of high concentrations of glycerol and glucose could significantly promote yeast proliferation under high-temperature conditions, so osmotic stress could not be regarded as a common factor to assist *Z. rouxii* in restoring proliferation under high-temperature conditions. Therefore, we selected 2% Tre, 20% Tre, and 20% Tre + 2% Glc as the experimental carbon source group to explore the effects of different concentrations of trehalose and the combination of trehalose and glucose on the high-temperature growth of *Z. rouxii*. As shown in Figure 1D, under the condition of 20% Tre + 2% Glc, the adaptation period of *Z. rouxii* under long-term high-temperature stress was 0–24 h, and the logarithmic period was 24–60 h.

### 3.2. Study on Metabolic Characteristics of Z. rouxii Under Long-Term High-Temperature Stress

#### 3.2.1. Carbon Source Absorption and Consumption

**Figure 2:**
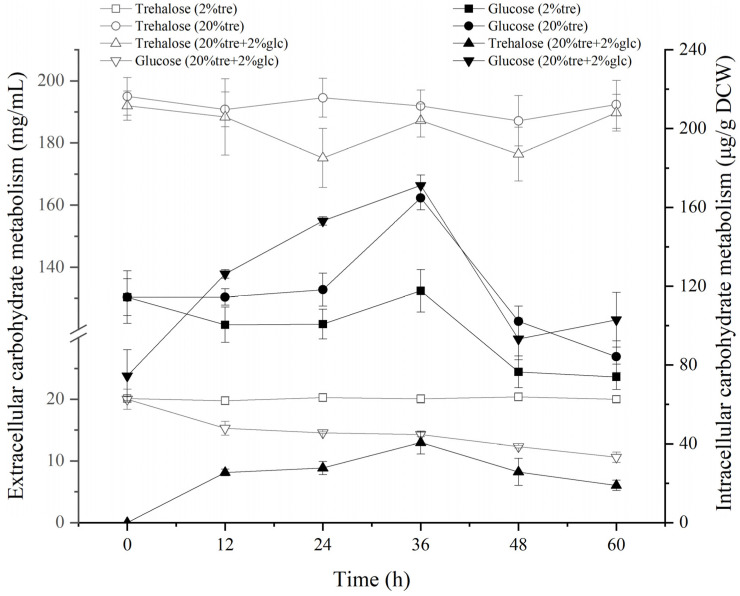
Changes of intracellular and extracellular trehalose and glucose concentrations in *Z. rouxii* grown at 40 °C. Note: The hollow point is the extracellular data point, and the solid point is the intracellular data point.

In order to explore the absorption and utilization of carbon sources by *Z. rouxii* under high-temperature conditions, we detected the changes of extracellular and intracellular trehalose and glucose concentrations. As shown in Figure 2, the extracellular trehalose concentration did not change much under 2% Tre and 20% Tre conditions, and no trehalose was detected in the cells, while the intracellular glucose concentration remained stable or even increased. We speculate that *Z. rouxii* mainly uses intracellular stored glucose as a carbon source, and extracellular trehalose is converted into glucose immediately after entering the cell. Under the condition of 20% Tre + 2% Glc, the extracellular glucose was continuously and stably absorbed, and the intracellular glucose reached 171.16 ± 5.28 μg/g DCW at 36 h, which was 2.31 times the amount at 0 h. Extracellular trehalose was also absorbed in a small amount, and intracellular trehalose was accumulated stably, reaching 40.62 ± 5.81 μg/g DCW at 36 h. Trehalose was not detected in the cells only with trehalose as the exogenous carbon source. It can be seen that the presence of exogenous glucose promoted the absorption and accumulation of trehalose by *Z. rouxii*. At 60 h, the carbon sources under the three conditions were not exhausted, so the growth difference of *Z. rouxii* under the three conditions was not caused by the lack of exogenous carbon sources.

#### 3.2.2. Detection of Stress-Resistant Carbon Metabolites

**Figure 3:**
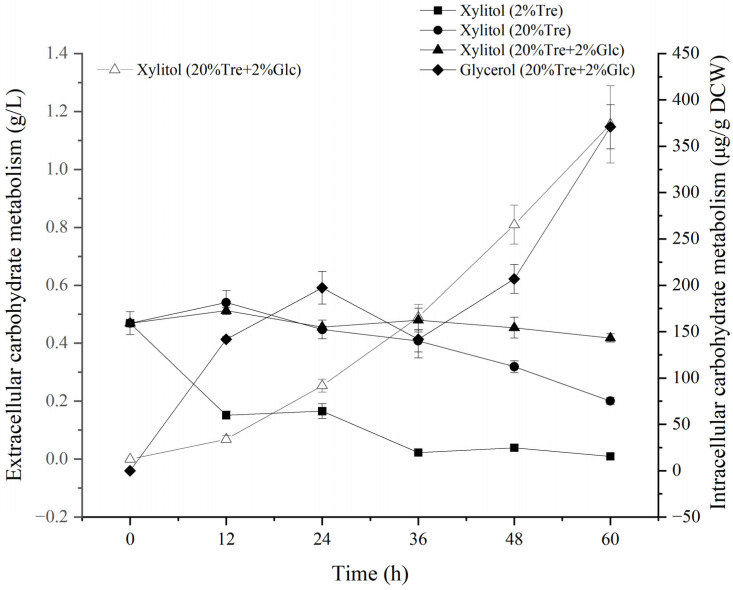
Detection of intracellular and extracellular stress-resistant carbon metabolites in *Z. rouxii* at 40 °C. Note: The hollow point is the extracellular data point, and the solid point is the intracellular data point.

Under high-temperature conditions, microorganisms usually produce stress-resistant metabolites to help themselves resist high-temperature stress. In order to explore whether *Z. rouxii* produces stress-resistant metabolites, we detected the concentration of intracellular stress-resistant metabolites. As shown in Figure 3, xylitol was detected in the cells of *Z. rouxii* under all three conditions. The initial concentration of xylitol under the three conditions of 2% Tre, 20% Tre, and 20% Tre + 2% Glc was equal, which was 159.05 ± 12.35 μg/g DCW. After 60 h, the concentration of xylitol was 15.47 ± 1.43 μg/g DCW, 75.15 ± 3.73 μg/g DCW, and 143.16 ± 4.46 μg/g DCW, respectively. Under the condition of 20% Tre + 2% Glc, the accumulation of extracellular xylitol was stable, reaching 1.15 ± 0.13 g/L at 60 h. Xylitol can not only be used as a carbon source, but also indirectly enter the PPP pathway to produce NADPH to resist ROS stress. The difference in xylitol consumption indicated that the amount of exogenous carbon source available to *Z. rouxii* under the three conditions was different, which may also suggest that *Z. rouxii* suffered different degrees of oxidative stress under the three conditions. Under the condition of 20% Tre + 2% Glc, *Z. rouxii* stably accumulated intracellular glycerol, reaching 370.89 ± 27.79 μg/g DCW at 60 h. Under the condition of 2% Tre and 20% Tre, glycerol was not detected. Glycerol is derived from glyceraldehyde 3-phosphate (GAP) in the EMP pathway, combined with the stable accumulation of extracellular xylitol under the condition of 20% Tre + 2% Glc, which indicates that there may be a surplus of carbon sources available to *Z. rouxii* under this condition.

#### 3.2.3. Detection of Organic Acids Related to Energy Metabolism

**Figure 4:**
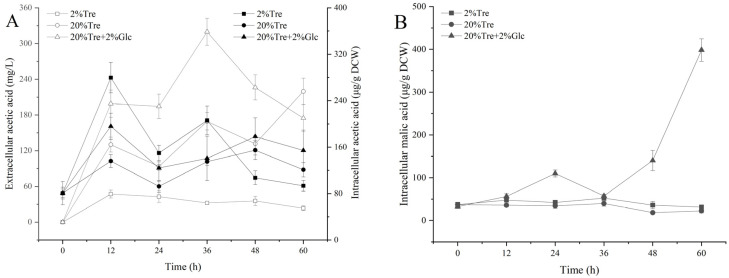
(**A**) Changes of intracellular and extracellular acetic acid in *Z. rouxii;* (**B**) changes of intracellular malic acid in *Z. rouxii.* Note: The hollow point is the extracellular data point, and the solid point is the intracellular data point.

Organic acid is an important product in the metabolic process of yeast. The change of its concentration reflects the change of metabolic pathway, the state of mitochondrial energy production, and the degree of acid stress. As shown in Figure 4A, the change trend of intracellular acetic acid concentration of *Z. rouxii* under the three conditions was basically the same, and the concentration remained stable after 12 h, all of which did not exceed 220 μg/g DCW. However, the extracellular acetic acid concentration of *Z. rouxii* under the three conditions was quite different, reaching 32.35 ± 2.98 mg/L, 168.42 ± 14.58 mg/L, and 319.70 ± 29.66 mg/L at 36 h, respectively. It can be seen that the amount of acetic acid produced by *Z. rouxii* under the three conditions is not the same, but *Z. rouxii* maintains the intracellular acetic acid content at a relatively stable level by effluxing acetic acid. Under the conditions of 2% Tre and 20% Tre, the intracellular malic acid content of *Z. rouxii* remained stable, and the maximum was not more than 40 μg/g DCW. Under the condition of 20% Tre + 2% Glc, malic acid was produced in large quantities after 12 h, and the highest concentration reached was 398.32 ± 26.28 μg/g DCW at 60 h. Acetic acid is one of the precursors of acetyl-CoA, and malic acid is an important intermediate metabolite in TCA cycle. We inferred that *Z. rouxii* produced more energy under 20% Tre + 2% Glc conditions, while *Z. rouxii* under 2% Tre and 20% Tre conditions may face the problem of insufficient energy supply.

### 3.3. Differential Analysis of Specific Gene Expression

#### 3.3.1. Carbohydrate Metabolite Synthesis Genes

##### Glycolytic Pathway

**Figure 5:**
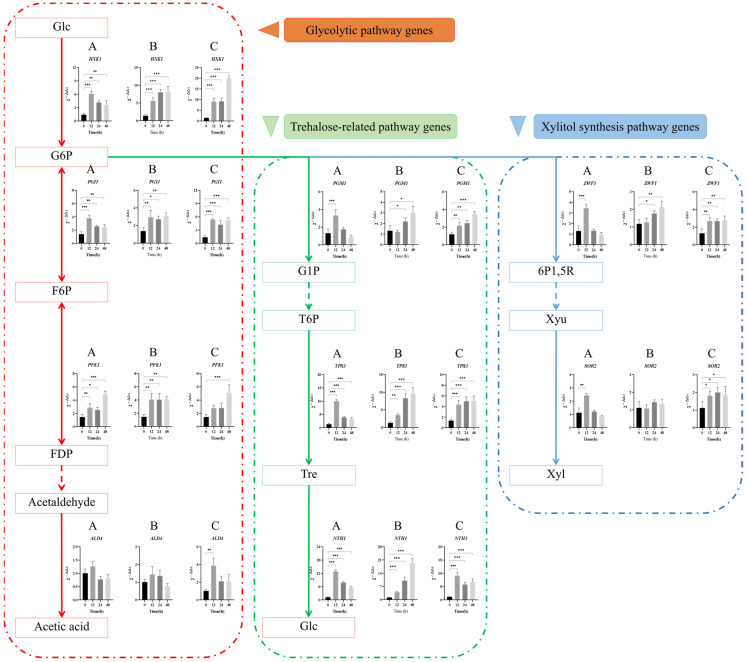
Relative transcriptional expression changes of carbohydrate metabolite synthesis gene mRNA. Note: The solid line arrow indicates one-step reaction, and the dotted line arrow indicates multi-step reaction. (**A**) 2% Tre; (**B**) 20% Tre; (**C**) 20% Tre + 2% Glc. Significance test: * *p* < 0.05; ** *p* < 0.01; *** *p* < 0.001.

The expression results of carbohydrate metabolite synthesis genes in *Z. rouxii* under long-term high-temperature stress are shown in Figure 5. The expression of aldehyde dehydrogenase gene *ALD4* was inhibited under 2% Tre and 20% Tre conditions, and the relative expression levels of hexokinase gene *HXK1*, a key rate-limiting enzyme of glycolysis, were significantly up-regulated by 3.6 and 8.3 times at 48 h, respectively, much lower than under 20% Tre + 2% Glc conditions (20 times). The expression levels of other genes such as phosphoisomerase *PGI1* and phosphofructokinase *PFK1* under 2% Tre and 20% Tre conditions were also lower than those under 20% Tre + 2% Glc conditions. The results showed that all genes in the glycolytic pathway were significantly up-regulated under the condition of 20% Tre + 2% Glc. The presence of glucose can promote the activity of key enzymes such as hexokinase and phosphate isomerase, thereby providing sufficient energy to help *Z. rouxii* adapt to long-term high-temperature environment.

##### Trehalose-Related Pathway

Under the condition of 2% Tre, the three genes *PGM1*, *TPS3*, and *NTH1* responded rapidly within 12 h, and were significantly up-regulated by 12.9, 3.1, and 10 times at 12 h, respectively, but were significantly down-regulated with the culture time. In particular, the expression of the key gene *PGM1* in the trehalose metabolic synthesis pathway was inhibited, and the gene expression was not active, which was consistent with the undetected results of trehalose at the metabolic level. Under 20% Tre and 20% Tre + 2% Glc conditions, all genes could maintain sustained expression, and the expression levels were significantly up-regulated.

##### Xylitol Synthesis Pathway

Under the condition of 2% Tre, the two genes responded rapidly at 12 h, and were significantly up-regulated by 3.5 and 2.4 times, respectively, but the expression of *ZWF1* and *SOR2* genes in the later stage of culture was inhibited, and the metabolic pathway was blocked. The expression level of 20%-Tre-condition gene *SOR2* did not change significantly. Under the condition of 20% Tre + 2% Glc, the expression of each gene could be maintained with the culture time, and the expression levels were significantly up-regulated. After 48 h of culture, the expression levels were significantly up-regulated by 2.8 and 1.9 times, respectively. The synthesis pathway of xylitol was not affected, which was consistent with the results of xylitol synthesis detected at the metabolic level under this condition.

#### 3.3.2. Transport Regulatory Protein Genes

**Figure 6:**
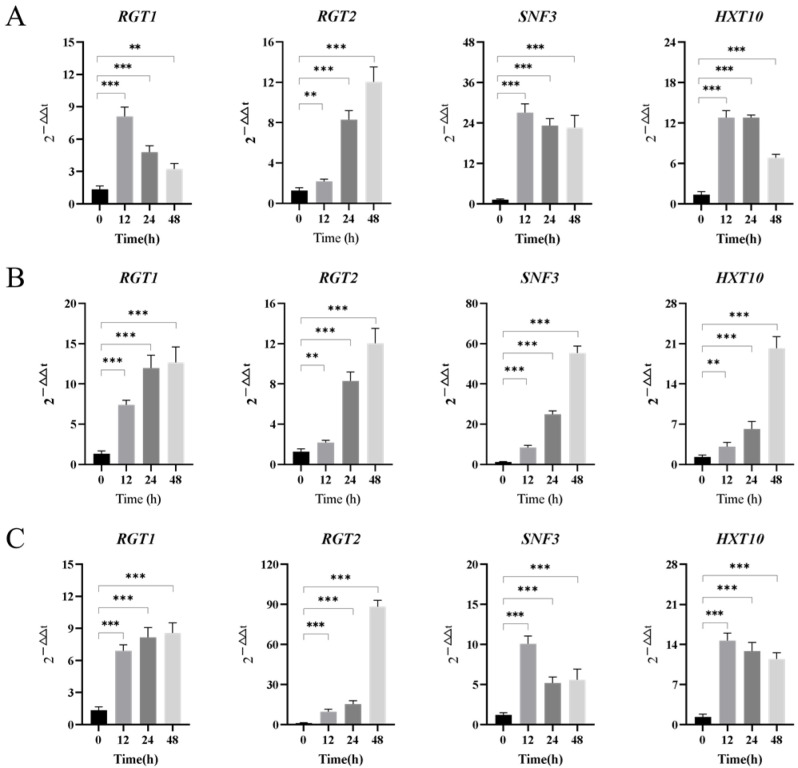
Relative transcriptional expression changes of transport regulatory protein gene mRNA. Note: (**A**) 2% Tre; (**B**) 20% Tre; (**C**) 20% Tre + 2% Glc. Significance test: ** *p* < 0.01; *** *p* < 0.001.

The expression results of transport regulatory protein genes in *Z. rouxii* under long-term high-temperature stress under three nutritional conditions are shown in Figure 6. All genes could respond quickly to the environment, and the expression level was significantly up-regulated. Under the conditions of 2% Tre (Figure 6A) and 20% Tre (Figure 6B), the expression of *RGT2* gene in *Z. rouxii* was up-regulated by 4.6 and 12 times, respectively, while the expression level of *RGT2* gene was up-regulated by 88.3 times under the condition of 20% Tre + 2% Glc (Figure 6C), which was much higher than under the previous two conditions. Under the condition of 20% Tre + 2% Glc, a high concentration of trehalose provides high glucose stimulation, promotes the expression of high-glucose-sensing and transport protein *RGT2* gene, and then transports glucose, which provides material and energy source for the growth and proliferation of *Z. rouxii*. This is consistent with the metabolic level. The expression levels of *SNF3* and *HXT10* genes increased under 20% Tre conditions. Under the condition of 20% Tre + 2% Glc, yeast extracellular glucose is consumed and metabolized to synthesize a variety of substances, while trehalose has no significant change under each condition, and cannot be metabolized into other substances to provide energy and nutrition for yeast.

#### 3.3.3. Stress-Resistant Global Regulatory Protein Genes

**Figure 7:**
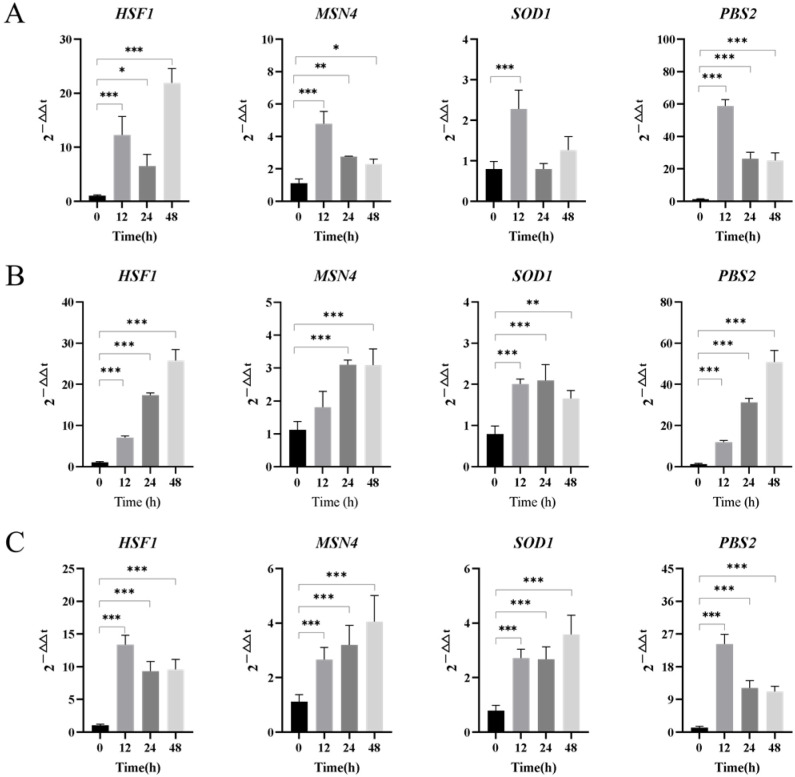
Relative transcriptional expression changes of stress-resistant global regulatory protein gene mRNA. Note: (**A**) 2% Tre; (**B**) 20% Tre; (**C**) 20% Tre + 2% Glc. Significance test: * *p* < 0.05; ** *p* < 0.01; *** *p* < 0.001.

In order to adapt to different degrees of heat shock stress during fermentation, *Z. rouxii* mainly adapts to environmental stress during fermentation by regulating the expression of some genes in the body [27]. The results of stress-resistant global regulatory protein gene expression in *Z. rouxii* under long-term high-temperature stress are shown in Figure 7. Under the condition of 2% Tre (Figure 7A), *Z. rouxii* responded quickly to stress, and the expression levels of the four genes were significantly up-regulated by 12.3, 4.8, 1.3, and 58.9 times at 12 h, respectively, but could not respond continuously. Under the conditions of 20% Tre (Figure 7B) and 20% Tre + 2% Glc (Figure 7C), increasing trehalose concentration or compounding 2% Glc can make stress-regulated genes continuously express. The *HSF1* gene was continuously up-regulated under the conditions of 2% Tre and 20% Tre, and remained stable under the condition of 20% Tre + 2% Glc, which indicated that the cells were subjected to a continuous increase in high-temperature stress in the medium with only trehalose, while the stress degree under the condition of 20% Tre + 2% Glc was relatively stable. In addition, it can be seen from Figure 7A,B that when trehalose was used as a single carbon source, the relative expression level of the osmotic stress signal transduction gene *PBS2* was significantly up-regulated compared with 0 h, up to 58.9 times and 50.9 times, respectively. Under the condition of 20% Tre + 2% Glc, the expression level was only up-regulated by 24.2 times. This indicates that the addition of exogenous trehalose will cause osmotic stress to *Z. rouxii* under the total synthetic medium with long-term high-temperature stress, but glucose can alleviate the osmotic stress caused by trehalose to *Z. rouxii*.

### 3.4. Analysis of Overall Gene Expression Differences

**Figure 8:**
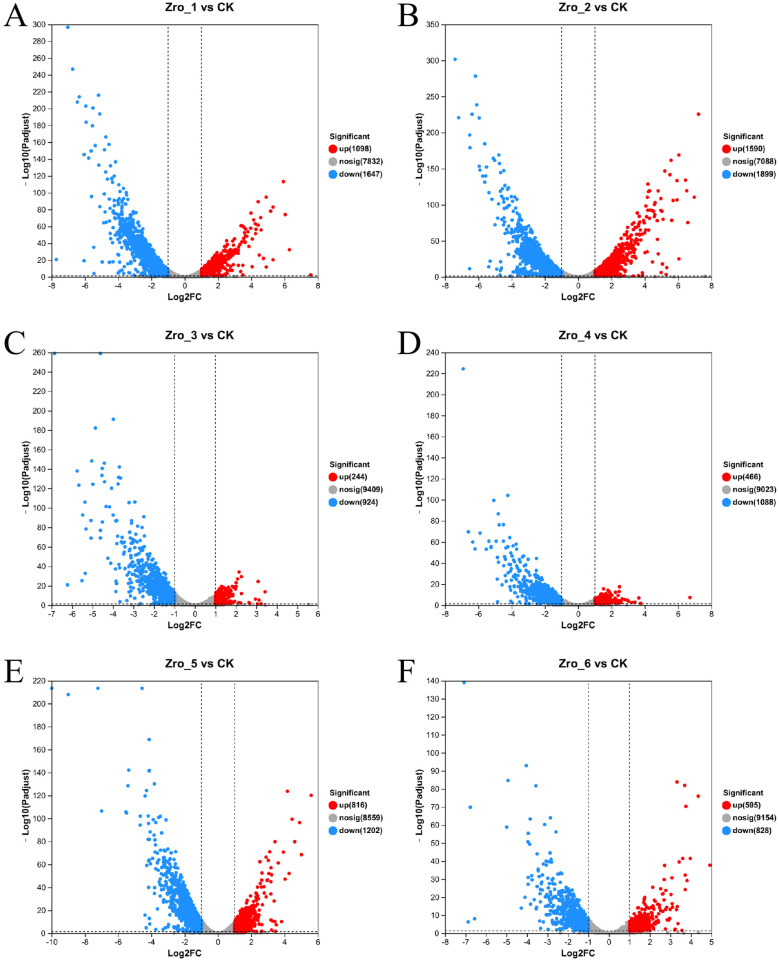
Volcanic map of expression difference of different samples. Note: (**A**) 2% Tre, 24 h; (**B**) 2% Tre, 48 h; (**C**) 20% Tre, 24 h; (**D**) 20% Tre, 48 h; (**E**) 20% Tre + 2% Glc, 24 h; (**F**) 20% Tre + 2% Glc, 48 h. The abscissa is the multiple of gene/transcript expression difference between the two samples, that is, the expression of the treated sample is divided by the expression of the control sample, and the ordinate is the statistical test value of the difference in gene expression change, that is, the *p* value. The larger the −log10 (Pvalue), the more significant the difference in expression, and the values of the horizontal and vertical coordinates are logarithmically processed. Each point in the figure represents a specific gene. The red point represents a significantly up-regulated gene, the blue point represents a significantly down-regulated gene, and the gray point is a non-significant difference gene.

In order to explore the molecular response mechanism of *Z. rouxii* to high-temperature stress, we analyzed the gene differential expression of *Z. rouxii* under high-temperature stress. The results are shown in Figure 8. Under the condition of only using Tre as carbon source, the total number of differential genes showed a time-series up-regulation trend. This indicates that *Z. rouxii* cannot effectively alleviate high-temperature stress when only using trehalose as a carbon source, and more genes need to be constantly adjusted to cope with this adverse environment. As shown in Table 1, compared with 2% Tre, the carbon source condition of 20% Tre reduced the number of up-regulated and down-regulated differential genes, but achieved the stability of the yeast living cell population, which may suggest that these genes play an important role in the high-temperature-adaptive proliferation of *Z. rouxii*. Under the condition of Tre + Glc as carbon source, the total amount of differential genes showed a downward trend in time series. This indicates that *Z. rouxii* may be more effective in alleviating high-temperature stress in the presence of glucose, so the adjustment of gene expression is relatively small, and this adjustment gradually decreases with time. The functional analysis of differential genes and KEGG pathway enrichment analysis also showed that *Z. rouxii* adopted different adjustment strategies for different functional genes under three conditions (Figures S2 and S3), which also confirmed that *Z. rouxii* was subjected to different degrees of high-temperature stress under different carbon source conditions, and its coping strategies were also different.

## 4. Discussion and Conclusions

High-temperature stress is one of the main stresses faced by yeast in industrial fermentation applications [28]. Trehalose is considered to be an important stress-protective substance for yeast to resist heat shock stress [29,30,31]. This study explored the mechanism of trehalose assisting *Z. rouxii* to resist high-temperature stress. The results showed that only using different concentrations of trehalose as a single carbon source could not promote the proliferation of *Z. rouxii*. Traditional literature has shown that trehalose can be used as a reserve carbon source for yeast to help it resist high-temperature stress [32,33]. However, the results of this experiment showed that trehalose alone could not restore the proliferation of *Z. rouxii* at high temperature. This indicates that trehalose cannot be used as the main carbon source for the growth and proliferation of *Z. rouxii* under high-temperature stress, and other types of carbon sources (such as glucose) are still needed to restore the growth of *Z. rouxii*.

The genes related to stress resistance and glucose metabolism in *Z. rouxii* (CTCC M 2013310) responded minimally to high temperature in the total synthesis medium with 2% Glc as carbon source [24]. The results of this study showed that although the *Z. rouxii* (CTCC M 2013310) in the 2% Tre medium also showed a decline trend and the metabolic activity was weak, the relative expression of the gene indicated that it could still respond quickly to the high-temperature environment. Increasing the concentration of trehalose can maintain the timing of gene expression. Combined with the results of metabolic detection, we found that although trehalose could not be used as the main carbon source of *Z. rouxii*, it could induce high-level gene expression as a gene activator of *Z. rouxii* under high-temperature stress without entering the cell. This also suggests that trehalose may affect *Z. rouxii* through an indirect signal transduction mechanism. It has been reported that trehalose can be associated with some signaling pathways [34,35], and our results further confirm its potential mechanism as an indirect factor. We speculate that this is because the absorbed glucose generates a stress-resistant metabolite in the cell, which reduces the stress pressure of the cell, so that some genes do not need to maintain high expression.

Compared with the culture conditions with Tre + Glc as the carbon source, the ribosome-related metabolic pathways were significantly enriched under the culture conditions with only Tre as the carbon source. This indicates that protein synthesis is more active in the medium with only Tre, which is consistent with the detection of the relative expression of amino acid biosynthesis genes (Figure S1). However, this also suggests that proteins in cells may be more damaged. Based on the fact that some literatures have shown that trehalose has the function of protecting intracellular proteins from heat shock [36,37], we speculate that under the condition of containing glucose in the medium, cells can absorb glucose and produce stress-resistant metabolites such as trehalose, making the intracellular protein conformation tend to be stable. Under the condition of only using Tre as a carbon source, extracellular trehalose cannot enter the cell, which also leads to the inability of intracellular proteins to be effectively protected. This speculation is also consistent with our detection results of intracellular stress-resistant metabolites. It can be seen that the intracellular trehalose of *Z. rouxii* under high-temperature conditions is mainly derived from the synthesis and metabolism of glucose, rather than direct extracellular absorption.

In general, yeast senses extracellular glucose levels through two paralogous glucose-sensing receptors, Rgt2 and Snf3, which sense high and low glucose levels, respectively [38,39]. However, the relative expression level of *RGT2* gene was significantly higher than that of *SNF3* gene under the condition of 20% Tre + 2% Glc carbon source. This result indicates that for *Z. rouxii*, this carbon source environment is not equivalent to a low-glucose-concentration environment, but is more likely to be regarded as a high-glucose-concentration environment. Therefore, we can further consider the question: under the condition of a high concentration of trehalose, is the high expression of *RGT2* gene due to trehalose directly as the whole-gene activator of *Z. rouxii*, or through some mechanism to ‘deceive’ the high-glucose-concentration-sensing system of *Z. rouxii*, so that it mistakenly identifies the presence of a high concentration of trehalose as a signal of high concentration of glucose? Wu et al.’s study revealed that the Snf3 receptor in *S. cerevisiae* can sense high concentrations of xylose and trigger gene expression similar to low concentrations of glucose [40]. However, there is no literature directly indicating that high concentrations of non-glucose carbon sources can directly trigger a molecular mechanism similar to that induced by high concentrations of glucose, thereby promoting the high expression of *RGT2* gene. Further studies on this mechanism may help us to better understand the complexity and flexibility of yeast metabolic regulation.

Under three different culture conditions, acetic acid and malic acid can be detected in the cell of *Z. rouxii*. In microorganisms, acetic acid is one of the precursors of acetyl-CoA [41], which can enter the TCA cycle to provide carbon source and energy for various biological reactions and cell maintenance; malic acid is an important intermediate metabolite of TCA cycle, and its content reflects the energy metabolism of microorganisms [42,43]. Under the three culture conditions, the concentration of intracellular acetic acid and malic acid gradually increased, and it was speculated that the energy produced by *Z. rouxii* under the three culture conditions gradually increased, which was also consistent with the change of the growth state of *Z. rouxii* under the three culture conditions from constant death-maintenance survival-proliferation recovery. It is worth noting that Guaragnella et al. showed that acetic acid stress is one of the important environmental stresses faced by yeasts [44]. The stability of intracellular acetic acid content and the continuous increase of extracellular acetic acid concentration in *Z. rouxii* also indicate that *Z. rouxii* continuously effluxes acetic acid to avoid its toxic effects on cells. During the culture time of this study, *Z. rouxii* did not decline due to excessive acetic acid content. Continued extension of the culture time may allow observation of this phenomenon.

**Figure 9:**
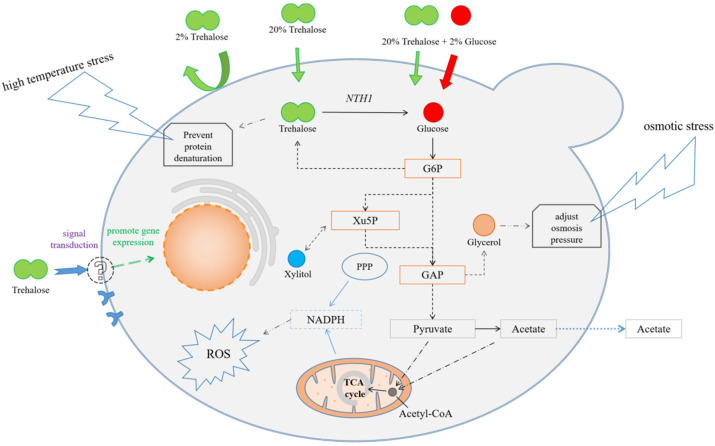
The model of trehalose assisting *Z. rouxii* to resist heat shock stress. Note: The black solid line arrow represents a one-step reaction; the black dotted arrow represents a multi-step reaction; the black dotted line arrow represents the anti-stress function of the substance; the blue solid arrow represents the NADPH production pathway; the blue dotted arrow represents the transport of acetic acid; the green arrow represents the absorption of Tre; the red arrow represents the absorption of Glc.

In summary, trehalose cannot be used as the main carbon source in the process of resisting high-temperature stress in *Z. rouxii*, but it can cause a variety of response mechanisms to cope with high-temperature stress (Figure 9). Exogenous trehalose is absorbed only with difficulty, and intracellular trehalose is mainly derived from the synthesis and metabolism of intracellular glucose. Trehalose can make *Z. rouxii* respond to high-temperature stress in a short time and up-regulate the expression level of global genes. Increasing trehalose concentration will maintain the sequential up-regulation of gene expression. High concentration of trehalose can be used to provide a high-glucose environment, promote the expression of high-glucose receptor gene *RGT2*, and cause yeast to absorb glucose in the presence of glucose in the medium. In summary, exogenous trehalose is a gene activator of *Z. rouxii* under high-temperature stress. The mechanism by which it assists *Z. rouxii* in coping with high-temperature stress is not by entering the metabolic pathway as the main carbon source, but by regulating the expression level of some genes of *Z. rouxii* through signal transduction.

## Figures and Tables

**Table 1:** The number of differentially expressed genes under different conditions.

Carbon Source Conditions	Detection Time Point	Total Number of Significant Difference Genes	The Number of Up-Regulated Genes	The Number of Down-Regulated Genes
2% Tre	24 h	2745	1098	1647
	48 h	3489	1590	1899
20% Tre	24 h	1168	244	924
	48 h	1554	466	1088
20% Tre + 2% Glc	24 h	2018	816	1202
	48 h	1423	595	828

## Data Availability

The original contributions presented in the study are included in the article/Supplementary Materials; further inquiries can be directed to the corresponding author.

## Supplementary Materials

The following supporting information can be downloaded at: https://www.mdpi.com/article/10.3390/jof10120842/s1, Figure S1: Relative transcriptional expression changes of nutrient-sensing network regulatory protein gene mRNA; Figure S2: Histogram of GO function annotation analysis of differential genes; Figure S3: Scatter map of KEGG enrichment of differential gene; Table S1: Related primer sequences and functional annotations.
