# Supplementary material for: Exogenous Trehalose Assists Zygosaccharomyces rouxii in Resisting High-Temperature Stress Mainly by Activating Genes Rather than Entering Metabolism
Source: J Fungi (Basel). 2024 Dec 5;10(12):842. doi: 10.3390/jof10120842 (PMC11677697; doi:10.3390/jof10120842)
Supplement: Supplementary file 1 [file jof-10-00842-s001.zip › Supplement.pdf]

Supplementary

Figure S1: Relative transcriptional expression changes of nutrient sensing network regulatory protein gene mRNA. Fig. A, 2%Tre; fig. B, 20%Tre; Fig. C, 20%Tre + 2%Glc.

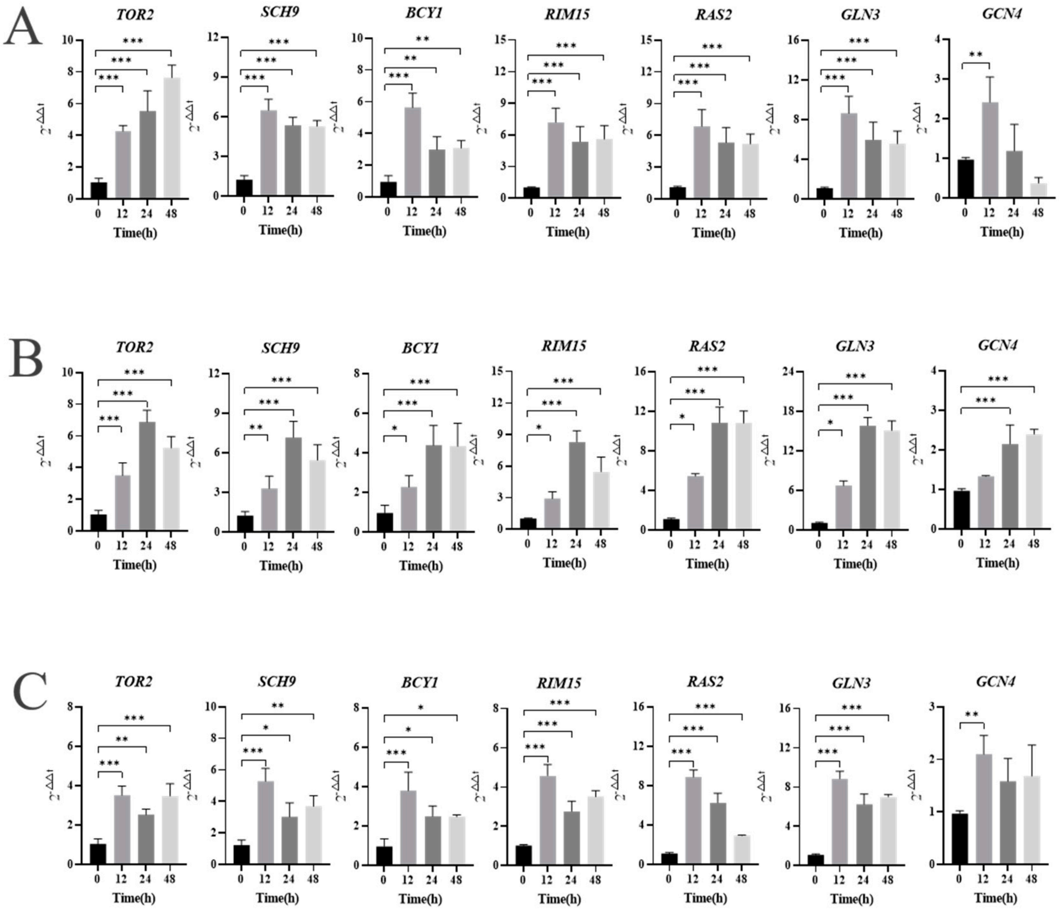

Figure S2: Histogram of GO function annotation analysis of differential genes. Figure. A-B：2%Tre；Fig. C-D：20%Tre ；Fig. E-F：20%Tre+2%Glc. BP: biological process; CC: cell components; MF: Molecular function.

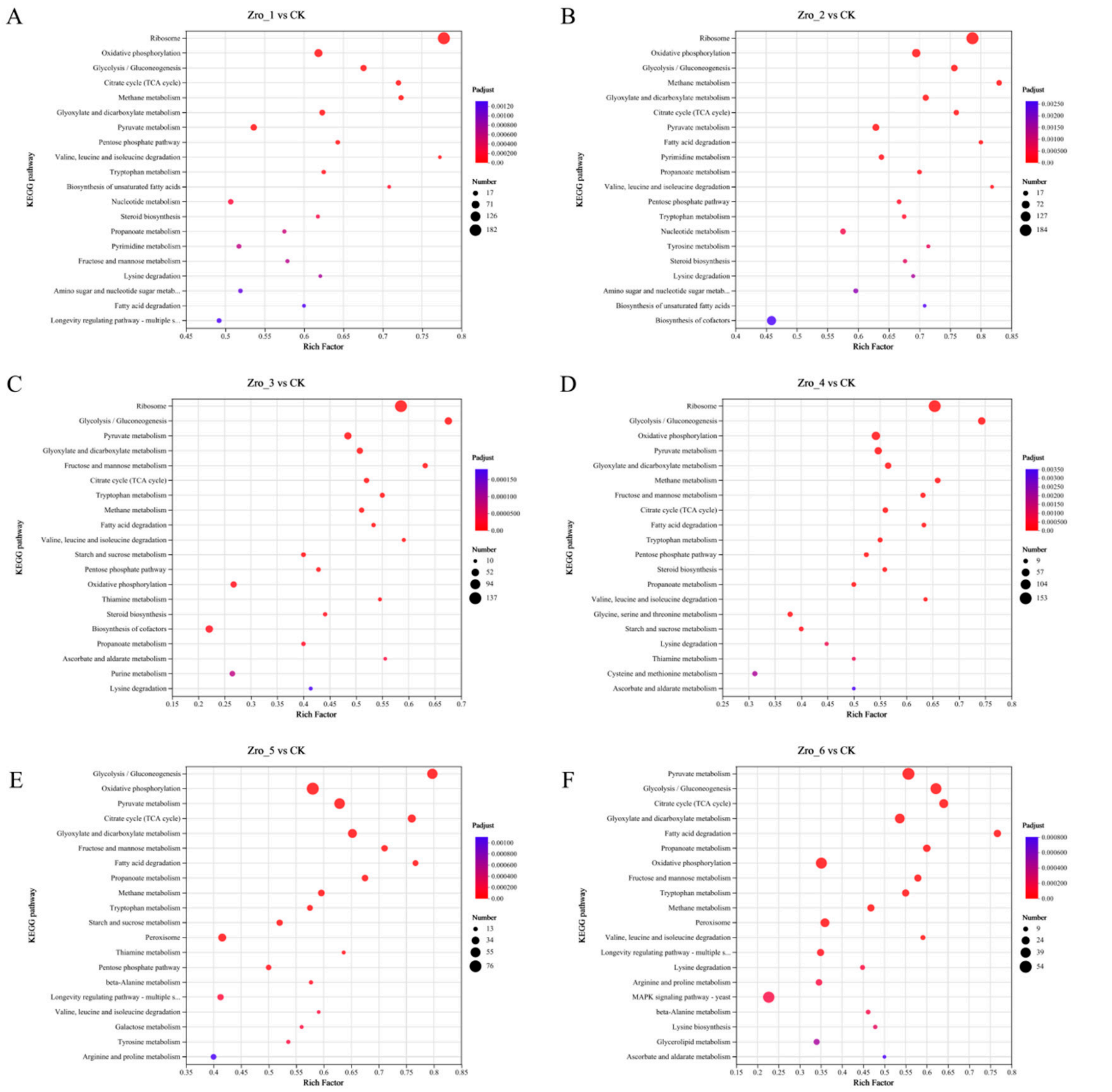

Figure S3 Scatter map of KEGG enrichment of differential gene. Figure. A-B：2%Tre；Fig. C-D：20%Tre ；Fig. E-F：20%Tre+2%Glc. The abscissa is the ratio of the number of differential genes annotated to the KEGG pathway to the total number of differential genes. The ordinate is the KEGG pathway. The size of the point represents the number of genes annotated to the KEGG pathway. The color from purple to yellow represents the significance of enrichment.

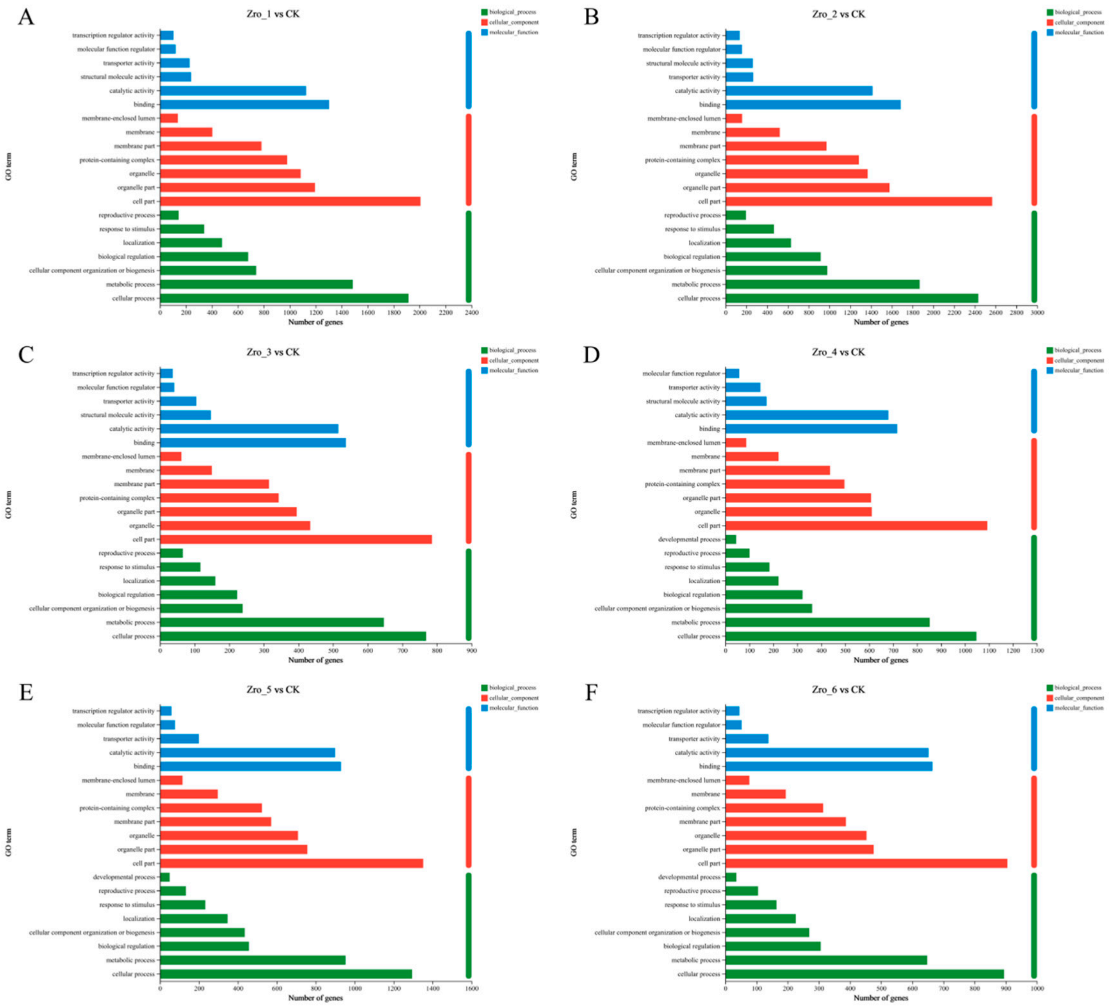

Table. S1 Related primer sequences and functional annotations

Note: After determining the generic name of the gene to be determined in Z. rouxii, we will search in NCBI and find its nucleotide sequence in "GenBank". Finally, we will use software Primer6 to synthesize the upstream and downstream primer sequences of the target gene and send them to the primer company for synthesis.

Primer name	Primer sequence(5'-3')	Functional annotations
ENO1-F	CGGTATGGACTGTG CTTCTTCTG	Housekeeping gene: a class of gene that is stably expressed in yeast cells of Z. rouxii, which is not

ENO1-R	GGATGGGTCGCTGTTAGGGTTCTT	affected by the treatment of research conditions, and is a reference gene that is consistently expressed between samples.
HSF1-F	TCTAAGCGGTCCTACTGAT	Heat shock transcription factor : Response to heat stress, responsible for regulating the expression of eukaryotic genes, by encoding HSPs chaperone gene transcription to mediate HSR
HSF1-R	TTGTTGAGTTCGGTGTCTT	
MSN4-F	TCATCAGCAGGAGATAGCA	Stress regulation response genes : encode stress regulation transcripts, involved in yeast cell stress response
MSN4-R	TCGGTGTAGATAGCGGTAA	
SOD1-F	CCAGATGGTGAAGTTAGACA	Oxidative stress regulatory protein gene : encodes cytoplasmic Cu / Zn superoxide dismutase, which catalyzes the decomposition of O2- to H2O2 and O2
SOD1-R	GGACGACAACGGTTCTAC	
PBS2-F	GCAACAACAACAACAGGAA	Osmotic stress signal transduction genes : encoding osmotic stress receptor protein, involved in osmotic stress signal transduction
PBS2-R	ATAATGATGACGACGGTGAT	
HXK1-F	GAAGTGATTGAGGTTGGTTAC	Hexokinase gene: encodes a hexokinase protein responsible for catalyzing the conversion of glucose to glucose 6phosphate.
HXK1-R	GGTTGCTGTTGTGGAGAA	
PGI1-F	GACTATCACTAACGCTAACAC	Phosphoisomerase gene: encodes a glucose phosphoisomerase protein that catalyzes the conversion of glucose-6-phosphate to fructose-6-phosphate.
PGI1-R	ACAGAGTAACGACCACCTA	
PFK1-F	TCTGCTGCTGTCATCTGT	Phosphofructokinase gene: encodes the fructose phosphokinase protein that catalyzes the conversion of fructose-6-phosphate and ATP to fructose-1, 6-diphosphate and ADP.
PFK1-R	ATGCCACTTCCGCTCTTA	
ADH2-F	ATCTCTGCCTCTACCAAGT	Alcohol dehydrogenase II gene: Ethanol dehydrogenase gene, encodes an alcohol

ADH2-R	TAACGACCTGCGATTAGAC	dehydrogenase protein and participates in ethanol biosynthesis.
ALD4-F	GGAATACGAACAACCAACAG	Acetaldehyde dehydrogenase gene: expressed using NADP or NAD as a coenzyme and inhibited by glucose.
ALD4-R	AACATCTTCTTCACGACCTT	
NTH1-F	CCGACATCATTGAGGAGTT	Trehalose hydrolase gene: codes trehalose hydrolase protein and catalyzes trehalose hydrolysis into glucose.
NTH1-R	CCATAGAGTAGTTGCCGATT	
PGM1-F	GGATTACCTGAAGACGAAGT	Phosphoglucomutase gene: catalyzes the conversion from glucose-1-phosphate to glucose-6- phosphate.
PGM1-R	TCCGCAATAATGGCAACA	
TPS3-F	TCGTGTTGTAGGTGTTGTT	trehalose-6-phosphate synthase gene: encodes trehalose synthase protein, catalyzing the conversion of trehalose-6phosphate to trehalose.
TPS3-R	CTGCTATATCTGTAGGTGTCA	
ZWF1-F	AGACGCAACTACCAGAATC	Glucose-6-phosphate dehydrogenase gene: encodes glucose-6-phosphate dehydrogenase protein, which is responsible for catalyzing the conversion of 6-phosphoglucose to 6phosphogluconic acid, while generating NADPH.
ZWF1-R	TCACCTTCAGCACCAGAT	
SOR2-F	GATAGAGTAGCCATTGAACCT	Xylose reductase gene: encodes a protein of Xylose reductase, catalyzing the conversion of Xylose to xylitol.
SOR2-R	CCAGAGACCAGCACCTTA	
RGT1-F	GAGGTAGAGTCACAGAGTTC	Glucose-responsive transcription factor gene: encodes glucose regulatory transcripts and participates in the regulation of glucose transporter HXT.
RGT1-R	ACAGCAGTAGCAGCAGTA	
RGT2-F	CTTCTGTAGTTCCA	Glucose high glucose receptor gene: encodes high

	CCATTG	concentration glucose receptor protein and
RGT2-R	ACCACCATATTGTTGACCTA	participates in glucose concentration signal transduction.
SNF3-F	ACCACTAAGGAGCAATCAAT	Glucose low glucose receptor gene: encodes low concentration glucose receptor protein and
SNF3-R	AAGAAGACACTGACAACACT	participates in glucose concentration signal transduction.
HXT10-F	GATAGCATTCCTTCCACTGA	Hexose transporter gene: encodes a hexose transporter protein and participates in the transport of hexose on the membrane.
HXT10-R	CCACAACATACACCAATACTG	
TOR2-F	ACCTTCCACTTCCACTATTG	The highly similar presumptive protein / phosphatidylinositol kinase is involved in actin
TOR2-R	AATCCTCACCAGCATTCTC	cytoskeleton translation initiation distribution and signal activation of meiotic phosphatidylinositol kinase homologues.
SCH9-F	GTCAACAACAACAACAACAG	Protein kinases are involved in the regulation of growth and play a major role in the coupling of cell size and cell division.
SCH9-R	GTAGAAGAAGCACCTCCAA	
BCY1-F	CCAAGAGGCAAGAGTCAG	The regulatory subunit of cyclic adenylate-dependent protein kinase (PKA), a
BCY1-R	CATCACCACTATCGTCATTG	central regulator of yeast metabolism and transcription.
RIM15-F	CTACCGAGAAGAATCCTGTTA	Glucose-repressor protein kinase gene : involved in the signal transduction of nutrients during cell
RIM15-R	CATCCTTACCTGTATTGTCATC	proliferation in response to the establishment of a nutrient-specific stationary phase.
RAS2-F	GACGATGGTGGTGCTTAT	GTP-binding protein gene : Farnesylation and palmitoylation required for regulating nitrogen
RAS2-R	CTGTGCTTGCCTGAGTATA	starvation response to sporulation and filamentous growth
GLN3-F	GTATCGTCAACCTC	Transcriptional activators regulated by nitrogen

	ATTATCG	metabolic repression (NCR) localization and
GLN3-R	GCCACAAGCATTA	activities regulated by nitrogen source quality.
	CACATC	
GCN4-F	GATGATCCTGTTGC	Transcriptional activators expressed by amino
	GATGA	acid biosynthetic genes in response to amino acid
GCN4-R	AGCGAGTCTCTGT	starvation are strictly regulated at both
	TCCAA	transcriptional and translational levels.
